# Chocolate Quality Assessment Based on Chemical Fingerprinting Using Near Infra-red and Machine Learning Modeling

**DOI:** 10.3390/foods8100426

**Published:** 2019-09-20

**Authors:** Thejani M. Gunaratne, Claudia Gonzalez Viejo, Nadeesha M. Gunaratne, Damir D. Torrico, Frank R. Dunshea, Sigfredo Fuentes

**Affiliations:** 1 School of Agriculture and Food, Faculty of Veterinary and Agricultural Sciences, University of Melbourne, Melbourne, VIC 3010, Australia; gunaratnem@student.unimelb.edu.au (T.M.G.); cgonzalez2@unimelb.edu.au (C.G.V.); mgunaratne@student.unimelb.edu.au (N.M.G.); damir.torrico@lincoln.ac.nz (D.D.T.); fdunshea@unimelb.edu.au (F.R.D.); 2 Department of Wine, Food and Molecular Biosciences, Faculty of Agriculture and Life Sciences, Lincoln University, Lincoln 7647, New Zealand

**Keywords:** sensory, physicochemical measurements, artificial neural networks, near infra-red spectroscopy

## Abstract

Chocolates are the most common confectionery and most popular dessert and snack across the globe. The quality of chocolate plays a major role in sensory evaluation. In this study, a rapid and non-destructive method was developed to predict the quality of chocolate based on physicochemical data, and sensory properties, using the five basic tastes. Data for physicochemical analysis (pH, Brix, viscosity, and color), and sensory properties (basic taste intensities) of chocolate were recorded. These data and results obtained from near-infrared spectroscopy were used to develop two machine learning models to predict the physicochemical parameters (Model 1) and sensory descriptors (Model 2) of chocolate. The results show that the models developed had high accuracy, with *R* = 0.99 for Model 1 and *R* = 0.93 for Model 2. The thus-developed models can be used as an alternative to consumer panels to determine the sensory properties of chocolate more accurately with lower cost using the chemical parameters.

## 1. Introduction

Chocolate is a semisolid suspension of fine particles made from sugar, milk powder, milk fat and cocoa in a continuous fat phase. The fruit of *Theobroma cacao* provides the cocoa solids and cocoa butter used for chocolate production. It is a confectionery product that evokes emotional stimuli upon consumption and activates the pleasure centers in the brain [1,2]. The main processing steps of chocolate manufacture are mixing, refining, conching, tempering, molding, and packaging. Particle size reduction takes place in the refining stage to obtain the optimum size of particles, which is important for the texture of chocolate [2]. Viscosity, mouthfeel, and consistency of chocolate are very important properties of chocolate, which are affected by rheological and textural characteristics. The processing conditions and compositions have an influence on the rheological properties of food [3]. To achieve the preferred rheological factors in chocolate with an acceptable texture, several production steps such as mixing, refining, conching, and tempering, are important. These processing parameters affect the viscosity of chocolate [4]. In order to obtain high-quality products, viscosity is considered an important physical parameter in producing chocolate and cocoa products, since it influences the textural properties of chocolate [4]. Moreover, acidity, sweetness, bitterness, color intensity, hardness, and smoothness are the most important parameters which affect the sensory perception of chocolate [5].

Bitterness can be caused by different chemical compounds, such as quinine hydrochloride and 6-n-propyl-2-thiouracil (PROP), and one of its functions is said to be the identification and avoidance of poison [6]. Sodium-ion (Na^+^) can be used as a reference to measure saltiness, and it impacts the ion channel of the taste receptor. Acids are responsible for causing sourness, and the hydrogen ion (H^+^) activates the taste receptor. Compounds which are responsible for sweetness vary extremely from simple carbohydrates to amino acids and artificial sweeteners [6]. “Umami” is a Japanese term that means “delicious” and is naturally found in food like meats, tomatoes, and mushrooms. It was identified through studies of monosodium glutamate (MSG) and is considered as a flavor enhancer [7]. 

Research and quality inspection of food products, such as sensory evaluation as well as the determination of physicochemical data is very time-consuming and labor-intensive and may require analytical techniques. Near infra-red (NIR) spectroscopy (NIRS) conforms to 750–2500 nm wavelength, which employs photon energy (hn) within the range of 2.65 × 10^−19^ to 7.96 × 10^−20^ J that is a promising technique which may overcome some of the drawbacks of traditional methods [8]. There are several advantages of using NIRS, including being a fast, non-destructive, non-invasive, and universally accepted technique [9]. Several studies have been conducted with NIRS and physicochemical data to quantify various analytes in foods [10]. Recent studies on food products, such as assessment of beer quality using computer vision algorithms, NIRS and machine learning algorithms [11], prediction of pH and total soluble solids in banana using NIRS [12], prediction of canned black bean texture using visible/NIRS [13], and discriminant analysis of pine nuts by NIRS [14] have been conducted. Applications of NIRS to cocoa and chocolate manufacture include quality control of cocoa beans [8], determination of biochemical quality parameters in cocoa [15], rapid determination of sucrose content in chocolate mass [16], and assessment of raw cocoa beans to predict the sensory properties of chocolate [17].

In this study, NIRS was used to gain chemical fingerprinting of chocolate produced using the five basic tastes. Predictive models based on artificial neural networks (ANN) were developed using Matlab^®^ R2018b (Mathworks Inc., Natick, MA, USA). Specific absorbance values of NIR spectra were used as inputs, while physicochemical data (pH, Brix, viscosity, and color) and sensory properties (basic taste intensity) of chocolate were used as targets. The objective of this study was to develop accurate models to predict the quality of chocolate based on chemical, physical, and sensory properties using NIRS and machine learning algorithms.

## 2. Materials and Methods 

### 2.1. Chocolate Samples

For this study, five different types of chocolate with basic tastes (bitter, salty, sour, sweet, and umami) were used. For the bitter sample, commercially available dark chocolate (70% cocoa) was used. Three concentrations each for the other four basic tastes were produced in the Sensory laboratory at The University of Melbourne, Australia. By tasting at a focus group discussion consisting of sensory professionals at The University of Melbourne, the final concentration of each taste was determined. They commented on the identification and the intensity of each flavor and the concentration which all the participants agreed, was used for final sample preparation. Therefore, salt (4 g), citric acid (0.5 g), sucrose (6 g) and MSG (3.5 g) were added to 100 g of melted compound milk cooking chocolate chips in order to produce salty, sour, sweet and umami samples respectively. These chocolate samples were used for sensory analysis, NIRS, and physicochemical analysis. 

### 2.2. Participants for Sensory Sessions

Panelists (*N* = 45) were recruited via email invitations from The University of Melbourne, Australia, who volunteered to participate in the sensory assessment of chocolate samples with basic tastes. The panelists had to sign a consent form before participating in the sensory session. The panelists received incentives (chocolate and confectionery products) as an appreciation for participating in the study. The experimental procedure was approved by the Ethics committee of the Faculty of Veterinary and Agricultural Sciences at The University of Melbourne, Australia (Ethics ID 1545786.2). 

### 2.3. Sensory Evaluation

Sensory sessions were conducted in individual booths in the sensory laboratory at The University of Melbourne. Each booth consisted of an integrated camera system controlled by a Bio-sensory application (App) designed for Android tablets (Google; Open Handset Alliance, Mountain View, CA, USA) developed by the sensory group from the School of Agriculture and Food, Faculty of Veterinary and Agricultural Sciences, The University of Melbourne [18]. The intensity of bitterness, saltiness, sourness, sweetness, and umami taste (0—low/7.5—medium/15—high) were assessed using a 15-cm non-structured continuous scale. The temperature of the serving/preparation room was 20 °C, while the temperature of the booths was controlled between 24 and 25 °C. Panelists were served with chocolate samples (two pieces from each taste; each square measuring 1 cm × 1 cm and weighing 7.3 g) on a tray in random order with specific 3-digit random numbers for evaluation. Panelists were asked to cleanse their palate using crackers and water in between all samples. 

### 2.4. pH, Total Soluble Solids (TSS) and Viscosity Measurements

For pH, five readings were taken from five pieces of chocolate (25 readings in total per sample). Initially, 10 g of chocolate was ground and mixed with 100 mL of distilled water and was allowed to settle for 20 min [19]. The pH of the supernatant liquid was measured (25 readings per each taste) using a calibrated bench-top meter (Sper Scientific Ltd., Scottsdale, AZ, USA) at room temperature (23–25 °C). The same method was modified to measure total soluble solids (Brix), and the supernatant liquid obtained from mixing chocolate with distilled water was used to measure the Brix value (25 readings per each taste) using a digital refractometer HANNA HI 96801 (Hanna Instruments Inc., Woonsocket, RI, USA) at room temperature (23–25 °C). In order to determine viscosity, chocolate was melted by microwaving 100 g for 1 min at 800 W [20]. A Brookfield DV1 viscometer with RV7 spindle at 50 RPM (AMETEK Brookfield, Middleboro, MA, USA) was used to measure the viscosity (6 readings per sample) of the melted chocolate at 28–30 °C. About 12–15 min were taken to obtain the measurements. 

### 2.5. Near infra-red Spectroscopy and Color Measurements

A handheld microPHAZIR^™^ RX Analyzer (Thermo Fisher Scientific, Waltham, MA, USA) was used to measure the NIR spectra of chocolate samples. The instrument was calibrated using a white background before obtaining the measurements as well as after 10–15 samples. This device can be used to measure the absorbance of wavelengths between 1600 and 2396 nm. 

In this study, 24 pieces from each type of chocolate were used to measure the spectra. Furthermore, three readings from the top surface and three from the bottom surface of the chocolate piece were obtained, providing 144 readings per sample. The obtained absorbance readings from chocolates manufactured with the five basic tastes were averaged separately in order to plot against wavelength. After considering all the pre-treatments, the second derivative values of absorbance were used for the analysis because they showed the best representation. The Unscrambler X ver. 10.3 (CAMO Software, Oslo, Norway) software was used to plot absorbance values with all wavelengths for the five chocolate samples.

The color parameters using the CIELab color scale were recorded using a StellarNet Inc. EPP2000 (EPP2000-UVN-SR) coupled with an SL1 Filter (StellarNet, Inc., Tampa, FL, USA). In the CIELab color scale, *L* represents lightness and ranges from 0 to 100, *a* depicts the red and green in the positive and negative values, respectively, and *b* represents yellow in the positive values and blue in the negative [21].

### 2.6. Statistical Analysis and Machine Learning Modeling

An analysis of variance (ANOVA) was conducted to assess significant differences (*α* = 0.05) of the physicochemical data between chocolates with different tastes using Minitab 2017 (Minitab Inc., State College, PA, USA). The mean values and standard deviation of the sensory, chemical, and physical parameters were also calculated and tabulated. 

A correlation matrix was also developed to identify the correlations between the sensory (basic taste intensities) and physicochemical properties (pH, Brix, color, and viscosity) of the chocolate samples using a customized code written in Matlab^®^ R2018b (Mathworks Inc. Natick, MA, USA).

**Figure 1:**
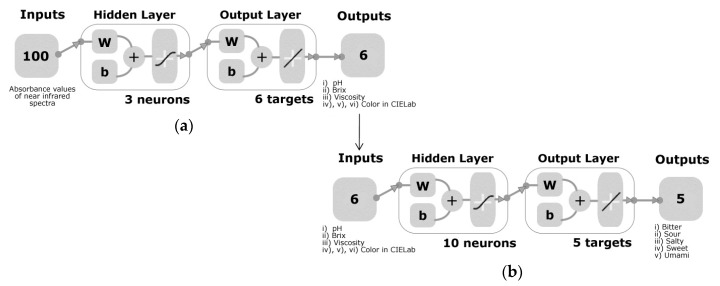
Diagrams with a two-layer feedforward network and tan-sigmoid function in the hidden layer, and a linear transfer function in the output layer for (**a**) Model 1 constructed with 100 inputs from near-infrared readings, three neurons and six targets related to physicochemical data of chocolate, and (**b**) Model 2 developed using six inputs obtained from the output of Model 1, ten hidden neurons and five targets related to basic taste intensities of chocolate. For the hidden and output layers, *w* = weights and *b* = biases.

As shown in Figure 1, two machine learning models were developed by testing 17 different ANN training algorithms using an automated customized code written in Matlab^®^ R2018b. The 17 algorithms were summed as two backpropagation using Jacobian derivatives, 11 backpropagation using gradient derivatives, and four supervised weight or biased training. An ANN fitting model (Model 1) with three hidden neurons was developed using the normalized NIR readings (−1–1) of chocolate as inputs and normalized values (−1–1) of physicochemical data (pH, Brix, viscosity, and color in CIELab) as targets. The whole NIR spectra (1596–2396 nm) was used to develop the model. The outputs of the Model 1 were used as inputs for the Model 2, while the sensory data (intensities of bitterness, saltiness, sourness, sweetness and umami taste) were used as targets. Both models were developed using a random data division, 70% (*n* = 84) of the samples were used for training, 15% (*n* = 18) for validation with a mean squared error (MSE) performance algorithm and 15% (*n* = 18) for testing using a default derivative function. Data division was randomized similar to Model 1, and 10 hidden neurons were used. From the 17 algorithms (data not shown), the best models corresponded to the Levenberg Marquardt algorithm in both Model 1 and Model 2. The coefficient of determination (R), slope (s), MSE and statistical significance (criteria; *p* < 0.05) were obtained. The p-value was calculated using CoStat ver. 6.45 (CoHort Software, Monterey, CA, USA) software. The performance of the models was evaluated based on the MSE. The most accurate model was selected from the above based on the R and MSE values for each stage (training, validation, testing and overall) and the slope of the overall model. Finally, a normality test (Jarque-Bera Test and DAgostino & Pearson Test) was conducted for the values of the error histogram using a customized code written in Matlab® R2018b to identify whether the errors were normally distributed.

## 3. Results

### 3.1. Sensory and Chemical Analysis 

The mean values and standard deviation of the results obtained from the sensory session (intensities of basic tastes), and physicochemical analysis are shown in Table 1. Significant differences (*p* < 0.001) were found in all the basic taste intensities (bitterness, saltiness, sourness, sweetness, and umami taste) for all five samples. According to the results of ANOVA conducted for the chemical parameters, there was a significant difference (*p* < 0.001) between the different chocolate samples in pH, Brix, and viscosity. 

### 3.2. Near Infra-Red Spectroscopy 

**Figure 2:**
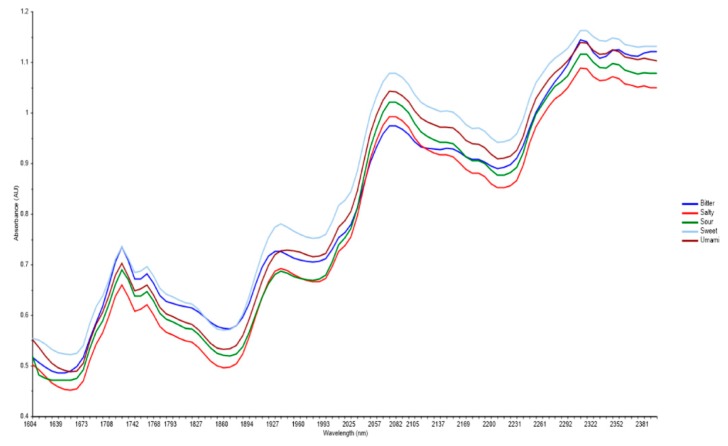
Curves for chocolate samples showing the absorbance (Au) values (y-axis) for specific wavelength (nm) values (x-axis) in the near infra-red spectra for each chocolate sample with basic tastes. A total of 144 absorbance readings per sample were taken, and the curves were drawn by using the mean values.

Figure 2 shows the graphs drawn using the mean values from the replicates of each type of chocolate. Different colors are used to signify each type of chocolate: dark blue for bitter, red for salty, green for sour, light blue for sweet and brown for umami. As seen in this figure, the peak values for all the chocolate samples are within the range of approximately 1700–2350 nm, in which compounds such as water, carbohydrates, proteins, sucrose, lactose, and fat can be found [22]. 

### 3.3. Multivariate Data Analysis

**Figure 3:**
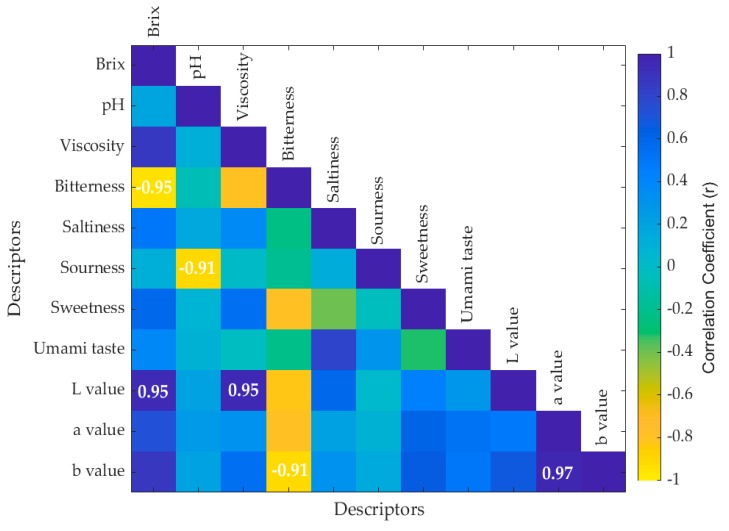
Results from the correlation matrix. Those with the values in the boxes represent the significant correlations (*p* < 0.05). The color bar represents the correlation coefficient (r) with the blue side being positive correlations and the yellow side the negative correlations. The x-axis and y-axis represent the descriptors.

The correlation matrix showing the values of the significant correlations (*p* < 0.05) between the chemical and sensory parameters of chocolate is shown in Figure 3. It can be observed that Brix was negatively correlated to bitterness (*R* = −0.95) and positively correlated to the *L* value (*R* = 0.95) while pH was negatively correlated to sourness (*R* = −0.91). There was also a negative correlation between bitterness and the *b* value (*R* = −0.91).

**Figure 4:**
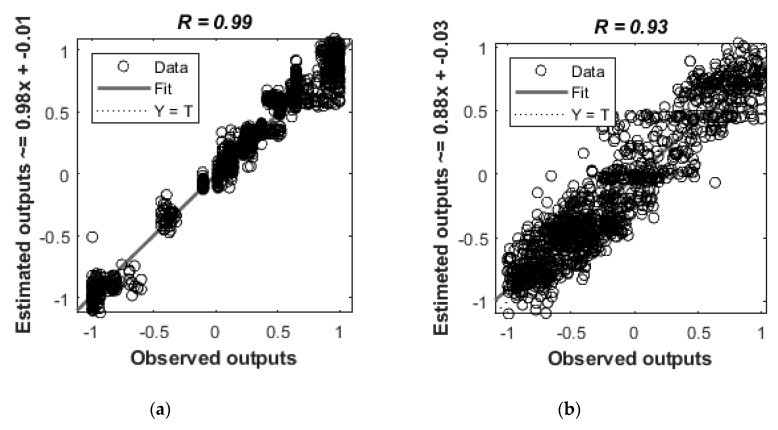
Results of artificial neural networks (ANN); (**a**) Model 1 using physicochemical data as targets and readings of the whole near-infrared wavelength range (1596–2396 nm) as inputs. (**b**) Model 2 using outputs of Model 1 as targets and sensory responses (basic taste intensities) as inputs. The observed values are shown on the x-axis and the estimated values on the y-axis.

Table 2 shows a summary of the statistical data obtained from both Model 1 and 2. Model 1 had a higher correlation coefficient (*R* = 0.99) for the overall stage (Figure 4a). It also had the same performance values for validation and testing stages (MSE = 0.01), which are an indication of no overfitting. The slope values of all the stages were closer to unity (*s* ~ 1). The overall correlation coefficient (*R*) of Model 2 was 0.93 (Figure 4b). Furthermore, it also had similar performance values for the validation and testing stages (MSE = 0.05) and values closer to the unity (*s* ~ 1) for the slopes of all the stages. Furthermore, data were normally distributed (*p* = 1) in the error histograms of Models 1 and 2 according to the Jarque-Bera Test and the DAgostino & Pearson Test. 

## 4. Discussion

The sour chocolate sample showed the lowest pH value according to the mean values obtained for all samples. This is due to the acidic pH obtained by adding citric acid to the chocolate. Furthermore, bitter chocolate had the lowest Brix value, which is an indicator of the total soluble solids (sugars) of the food. These results are also shown in the correlation matrix (Figure 3), which is discussed later in this paper. Moreover, the bitter chocolate indicated the lowest result for the *L* value of the color measurements, which shows that it was the darkest sample when compared to others because 0 indicates black and 100 shows white in the *L* value of the CieLab scale [23]. 

The NIR curves for all five chocolate samples exhibiting bitter, salty, sour, sweet, and umami taste profiles showed similar trends. The main ingredients of chocolate are cocoa liquor, cocoa butter, sugar, milk powder, and milk fat. These compounds contain peptide and carbohydrate bonds and hence, contribute to the peaks of spectral [24]. According to Figure 2, peaks were observed at 1728 nm and 1761 nm, which match the C-H bond of carbohydrates which is similar to the profile of cocoa butter [24]. The peak around 1940 nm is the water content in the chocolate which may be the water contained in the ingredients used for chocolate production [22]. The NIR curves also showed peaks around 2100 nm and 2310 nm, which show the presence of protein, mainly from the milk powder and cocoa butter in the chocolate. The peak around 2080 nm due to the absorbance of O–H bond in sucrose indicates the presence of sugar in chocolate. Moreover, the peaks around 1759 nm, 2310 nm, and 2343 nm indicate the presence of fats in the chocolate [24]. In food science, qualitative methods play an important role in NIRS analysis based on physicochemical data [25,26]. Hence, these qualitative methods were used in this study to determine the available compounds in the chocolate samples. 

Brix is an indicator of the total soluble solids (sugars) in a food product, where the sweetness is high when the value increases [27]. This was consistent with the correlation matrix presented in this study, which showed a negative correlation between Brix and sensory bitterness. The pH is an indicator of acidity in a sample [27]; therefore, sourness decreases with pH as basicity increases, and this was also in accordance with this study, as seen in the correlation matrix as pH had a negative correlation to sourness. Furthermore, bitterness showed a negative correlation to the *b* value, which indicates the reduction of yellow color. This complied with the findings showing that brown color is associated with chocolate products [28]. Hence, the instrumental measurements were correlated to the sensory characteristics. Recently, NIRS applications have been extended by the development of many calibration models along with physicochemical data. In this study, two models with high accuracy were developed using NIRS results, physicochemical data, and sensory intensities to predict the quality of chocolate. These can be used for qualitative or quantitative analysis of food products, and the main methods used for quantitative analysis are principal component regression, step multiple linear regression, partial least squares and ANN [29,30,31,32,33,34]. In the present study, ANN machine learning models were developed out of the above-mentioned methods.

Chocolate has also been a target for research using NIRS for several purposes. Nutritional parameters have been measured in chocolate using near-infrared diffuse reflectance spectroscopy and neural networks [24]. Similarly to the present study, that study also used ANN models as an alternative to time-consuming chemical methods of analysis. According to the results of the present study, the regression models developed using physicochemical data (pH, Brix, viscosity, color in L*a*b) showed high accuracies with a high correlation coefficient (*R*^2^ = 0.99), meaning that better predictions on chemical factors can be obtained using the developed models. 

Both models in this study showed similar MSE values for validation and testing stages, which means that there was no overfitting [35,36]. Due to the high accuracy of Model 2, which can be used to predict the sensory properties of chocolate using chemical and physical parameters (color), it may be used as a fast-screening method to determine the basic taste intensities of chocolate. Using Model 1, we can predict the physicochemical data (pH, Brix, viscosity, and color) of chocolate samples. Furthermore, the sensory properties (intensity of bitterness, saltiness, sourness, sweetness, and umami taste) of chocolate can be predicted using Model 2. Furthermore, this does not require the measurements of NIR but instead, requires the chemical and physical parameters which will be a lower cost compared to near infra-red spectroscopic measurements, considered as a high-cost technique by some researchers [37,38]. Moreover, if an investment can be made on a NIR device, it may be used to obtain all the chemical fingerprinting, physicochemical and the sensory data, which may reduce the time and cost of analysis. 

Understanding of food products over regression models for physicochemical data and sensory properties of food can be done using predictive modeling. Research also has been done to assess beer chemometry using novel techniques such as non-linear methods by developing predictive models using PLS and ANN [39]. It showed better predictive models when developed using ANN when compared to PLS. This was also found in the study from Cen and He [29], where they stated that ANN gives better results for some non-linear data than linear approaches. Furthermore, PLS can only generate models for training and validation stages, while ANN can generate for all four stages, which may also be used to find any signs of over or underfitting [11].

NIRS has several benefits when used in food analysis. The spectral measurement takes only 15–90 seconds; hence, NIRS is considered a rapid technique [40]. Furthermore, several samples may be analyzed using one spectral measurement, which is very beneficial in terms of analyzing various indexes. The physical state of the sample also does not matter for NIRS and can be directly tested in the sample chamber. NIRS is also a non-destructive method that is free of chemicals, which is another advantage of this technique [29]. There are few disadvantages in NIRS, such as showing insensitivity to lower concentrations (below 0.1%), the technique being an empirically based quantitative tool and necessity of careful observation for accurate results [41].

## 5. Conclusions

In this study, the use of NIRS for the prediction of chocolate quality based on chemical, physical, and sensory parameters was reinforced. NIRS and machine learning algorithms can be used to develop accurate prediction models to assess the quality of chocolate. The developed models can be potentially used as a rapid method to obtain physicochemical data and screen the intensity of basic tastes in chocolate based on sensory properties using a minimal amount of laboratory instruments and labor. Further studies may be conducted to improve the quality of models using other physicochemical measurements and sensory properties, which were not considered in this study.

## Figures and Tables

**Table 1:** Mean and standard deviation values of the sensory, chemical, and color data of the chocolate samples used for this study.

Sample	Bitterness	Saltiness	Sourness	Sweetness	Umami	
**Bitter**	10.16 ± 3.62 ^a^	2.25 ± 2.91 ^c,d^	2.35 ± 3.40 ^b,c^	4.31 ± 3.34 ^c^	3.28 ± 4.13 ^c^	
**Salty**	3.15 ± 3.90 ^b^	13.37 ± 2.25 ^a^	4.17 ± 4.39 ^b^	5.12 ± 3.69 ^c^	6.40 ± 4.70 ^a,b^	
**Sour**	2.17 ± 2.85 ^b,c^	3.93 ± 3.82 ^c^	9.54 ± 4.29 ^a^	9.00 ± 3.61 ^b^	4.79 ± 3.77 ^b,c^	
**Sweet**	0.99 ± 2.35 ^c^	1.84 ± 2.48 ^d^	1.15 ± 2.05 ^c^	11.95 ± 3.39 ^a^	2.56 ± 3.22 ^c^	
**Umami**	2.82 ± 4.07 ^b,c^	7.02 ± 3.17 ^b^	3.31 ± 3.67 ^b,c^	7.85 ± 4.45 ^b^	7.43 ± 5.25 ^a^	
**Sample**	**pH**	**Brix**	**Viscosity (cP)**	***L* Value**	***a* Value**	***b* Value**
**Bitter**	6.40 ± 0.07 ^c^	3.90 ± 0.34 ^d^	13680 ± 2319 ^d^	40.73	12.19	4.37
**Salty**	6.56 ± 0.07 ^b^	6.27 ± 0.89 ^a^	23443 ± 618 ^a^	65.21	20.47	23.05
**Sour**	5.42 ± 0.01 ^d^	5.64 ± 0.28 ^c^	19360 ± 444 ^b^	53.57	22.55	23.95
**Sweet**	6.91 ± 0.40 ^a^	6.20 ± 0.19 ^a,b^	23600 ± 664 ^a^	60.94	23.49	26.02
**Umami**	6.90 ± 0.05 ^a^	5.82 ± 0.58 ^b,c^	16747 ± 674 ^c^	53.19	30.40	32.30

a–d Means with different letters for each parameter indicate significant differences (*p* < 0.05) by Tukey’s studentized Range (HSD) test. ± standard deviation of mean values is stated. Bitterness, saltiness, sourness, sweetness, and umami taste were obtained from a 15-point continuous scale.

**Table 2:** Statistical data showing the stage, number of samples, correlation coefficient (*R*), performance based on mean squared error (MSE), and slope for Model 1 and 2.

Stage	Samples	*R*	Performance (MSE)	Slope
**Model 1**				
**Training**	84	0.99	0.001	0.98
**Validation**	18	0.99	0.01	0.99
**Testing**	18	0.99	0.01	0.97
**Overall**	120	0.99	0.01	0.98
**Model 2**				
**Training**	84	0.94	0.05	0.88
**Validation**	18	0.93	0.05	0.91
**Testing**	18	0.93	0.05	0.86
**Overall**	120	0.93	0.04	0.88
